# Prevalence and Risk Factors for Pterygium in Rural Older Adults in Shandong Province of China: A Cross-Sectional Study

**DOI:** 10.1155/2014/658648

**Published:** 2014-06-12

**Authors:** Wanzhen Jiao, Chengchao Zhou, Ting Wang, Shaoyuan Yang, Hongsheng Bi, Liping Liu, Yan Li, Lihua Wang

**Affiliations:** ^1^Department of Ophthalmology, Shandong Provincial Hospital Affiliated to Shandong University, Jinan 250021, China; ^2^Institute of Social Medicine and Health Service Management, School of Public Health, Shandong University, Jinan 250012, China; ^3^Shandong Eye Hospital, Shandong Eye Institute, Shandong Academy of Medical Sciences, Jinan 250021, China; ^4^Department of Ophthalmology, Qianfoshan Hospital Affiliated to Shandong University, Jinan 250014, China; ^5^Eye Hospital, Shandong University of Traditional Chinese Medicine, Jinan 250355, China

## Abstract

To investigate the prevalence and risk factors for pterygium in rural older adults in Shandong Province, eastern China, a population-based, cross-sectional study was performed from April to July 2008. By means of cluster random sampling methods, a total of 19,583 people aged 50 years or above were randomly selected from four rural counties. Out of 19,583 people, 1,767 residents were excluded mainly because they were migrant workers when this study was performed. Finally, 17,816 (90.98%) people were included as eligible subjects. They received a comprehensive eye examination and a structured questionnaire voluntarily. Patients with pterygium were defined as having pterygium at the time of survey or pterygium surgery had been performed. 1,876 people were diagnosed as pterygium, either unilateral (1,083) or bilateral (793), which is equivalent to a prevalence of 10.53% (95% CI, 10.08–10.98). The multivariate logistic regression analysis showed that pterygium was independently associated with older age, areas, outdoor time, educational level, and use of hat and/or sunglasses. The prevalence of pterygium increased with age and hours spent under sunshine per day. Meanwhile, the higher the educational level and the more use of hat and/or sunglasses, the lower the pterygium prevalence.

## 1. Introduction

Pterygium is a common external ocular disease with prevalence ranging between 0.7% and 33% globally [1]. The disease is described as a wing-shaped, oriented fibrovascular lesion that crosses the nasal or temporal limbus and can result in obvious cosmetic problems, significant astigmatism, and visual impairment or blindness due to interference with visual axis. Surgery is warranted for pterygium approaching the central part of the cornea. Unfortunately, the recurrence rate after the surgery is between 30% and 90% [2]. Numerous theories regarding the pathogenesis of pterygium included genetic, environmental, infective, and immunological factors [3]. Although the hypothesis implicating ultraviolet radiation (UVR) as a risk factor has been well studied in population-based studies, case-control studies, and laboratory studies, the definitive cause of pterygium is not well understood [4]. Treatment for pterygium has been improved in recent years to reduce the recurrence rate; for example, the recurrence rate after pterygium excision with limbal conjunctival autograft was lower than that of bare sclera, bulbar conjunctival autograft, or intraoperative mitomycin C application [2]. There were a few population-based studies of pterygium in different regions of China [5–10]. However, the prevalence of and risk factors for pterygium in rural areas of east China, for example, counties of Shandong Province, have not been sufficiently investigated. This study was performed to provide informative resources of pterygium in rural residents aged 50 years or above in Shandong Province.

## 2. Methods

This study was part of a population-based study of visual impairment and blindness in people aged 50 years or above in rural Shandong Province from April 2008 to July 2008 [11]. Shandong Province (northern latitude of 34°22.9′–38°24.0′, eastern longitude of 114°47.5′–122°42.3′) is located along the east coast of China, south of the lower reaches of the Yellow River, and covers an area of 157,100 km^2^, with a total population of about 95.79 million. Shandong Province has a typical temperate monsoon climate with four distinct seasons, hot and rainy summer, and cold and dry winter. The mean annual rainfall is approximately 676.5 mm. The mean temperature is 11–14°C. The yearly mean total global radiation is 481–540 KJ/cm^2^ with 2290–2890 hours of sunshine per year. This survey was approved by the Ethics Committee of Shandong Provincial Health Bureau and was in accordance with the tenets of the Declaration of Helsinki Principal. All participants signed consent forms at the local examination sites. Their examinations and treatments were free.

### 2.1. Sampling

**Figure 1:**
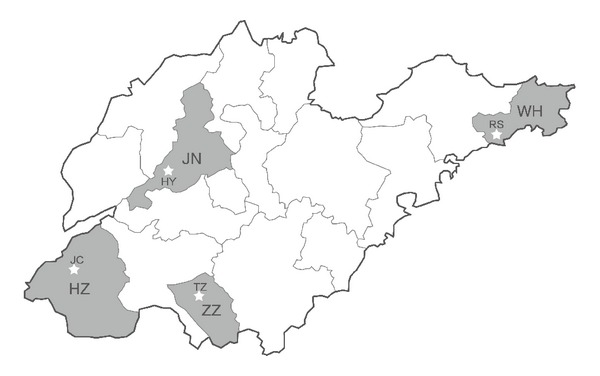
The geographical map of Shandong Province and the geographic location of the study site (*☆*). JN = Jinan City, HY = Huaiyin District; WH = Weihai City, RS = Rushan County; ZZ = Zaozhuang city, TZ = Tengzhou County; HZ = Heze City, JC = Juancheng County.

There are 88 counties or county-level cities in Shandong Province. According to data of per capita gross domestic product (GDP), all of these counties were divided into three categories: high GDP (≥¥5000), medium GDP (¥3000–¥5000), and low GDP (≤¥3000). Then, cluster random sampling method was used to select counties from each category. Consequently, Rushan County, Tengzhou County, Huaiyin District, and Juancheng County, representative of the different levels of socioeconomic development, were identified as the study areas (Figure 1). Health administrative departments in these four counties were asked to provide the demographic data and village registers. On the basis of this data, we defined the basic sample unit (BSU), which has a population of approximately 1,000 residents including all age groups, according to the following principles: (1) if the population in a village was equivalent to or close to 1,000, the village was presented as one BSU; (2) if the population in a village exceeded 1,500, we subdivided the village into several parts and randomly selected people from these parts to establish one BSU with a population of approximately 1,000; (3) if the population in a village was less than 500, we combined the population of the village with that of a neighboring village of similar size to establish one BSU. Then we numbered BSUs and ascertained study sites through simple random sampling.

For the purpose of determining the sample size, the prevalence of blindness was estimated to be 2.65% [27, 28]. We assumed an allowable error of 25%, a confidence interval (CI) of 95%, a response rate of 85%, and a design effect of 1.5 (accounting for inefficiencies associated with the cluster sampling design). As a result, a sample of the total number of the subjects required was 15,940. Depending on the percentage of population aged 50 years or above in each BSU, 76 BSUs were finally selected in four counties in order to reach the sample size for this survey.

### 2.2. Presurvey Stage

One month before the formal investigation, we determined all staff that would take part in the field investigation. Strict training for the staff was organized, including familiarization with the research objectives, the process of field investigation, eye examination skills and diagnostic criteria, and instructions for completion of the questionnaire. Then, we conducted a survey in a village which has a population of approximately 500 residents as a rehearsal, the results of which were not included in the present study. The survey included approximately 180 residents aged 50 years or above from Zhangqiu County near Jinan City. Based on this survey, we addressed some issues in the eye examination procedures and the organization processes. In addition, we determined the repeatability of intra- and interinvestigator by calculating Kappa values. The formal investigation was not initiated until the Kappa value was more than 0.7.

### 2.3. Questionnaire and Eye Examination

The present study was conducted by four teams, and each team included two groups. The enumeration group consisted of two trained field investigators who took charge of check-in and questionnaire investigation. Another group was composed of ophthalmologists and optometrists to carry out visual acuity (VA) testing and eye examination. Teams were provided with E Standard Logarithm Eyesight Table (SLD-3, Weixinyiao Science and Technology Co., Beijing, China), slit-lamp microscope (Topcon SL-2F, Topcon, Tokyo, Japan), automatic refractor (KR8800, Topcon, Tokyo, Japan), direct ophthalmoscope (YZ11D, 66 Vision Tech, Suzhou, China), and penlight.

The lists of residents aged 50 years or above were provided by the local administrative department. The enumeration group made contact with residents and tried to confirm whether they would participate in this study. They visited all people on the lists and explained the purpose and significance of this investigation. Residents who did not present at the examination site were revisited by staffs in the enumeration group in order to encourage participation in the study. Participants received eye examination at local test sites. Those elderly or physically disabled residents were offered door-to-door examination by doctors using portable equipment which comprised E standard logarithmic visual acuity chart, penlight, and direct ophthalmoscope. The response rate was required to be more than 85% to achieve the goal. Ophthalmologists checked participants' visual acuities, eyelids, eye globes, pupillary reflex, anterior segment, and fundus of the eyes. A structured questionnaire was conducted by trained field investigator for demographic data (name, gender, age, nationality, education, and socioeconomic status), living habits (alcohol intake, smoking, outdoor time per day, and wearing hat and/or sunglasses when outside), and comprehensive medical or eye surgery history. Educational level was obtained from the question “How many years have you been in school?” In the present logistic regression analysis, we classified answers into two categories: primary school and below (<6 years) and junior school and above (≥6 years). Alcohol intake and cigarette smoking were categorized as “Never,” “Current,” and “Past.” Duration of alcohol intake and the frequency of smoking per day were also obtained. The subject's outdoor time was evaluated from the question “How many hours do you spend under sunshine every day?” The responses were combined into two groups as “less than four hours” and “equivalent to or more than four hours.” The staff also asked the participants if they wore a hat and/or sunglasses when outdoors (Yes or No). The comprehensive medical history and eye surgery history were recorded based on the participants' self-report.

### 2.4. Definition of Pterygium

Pterygium is a radially oriented fibrovascular lesion crossing the corneoscleral limbus and encroaching on the clear cornea. Patients with pterygium in the present study were defined as (1) having pterygium at the time of survey or (2) having pterygium surgery performed and no pterygium at the time of survey.

### 2.5. Data Analysis

The collected data were transferred into the computer using Epi Info software (version 3.3, Centers for Disease Control and Prevention, Atlanta, GA, USA). The prevalence rate and 95% CI of pterygium were calculated. The Chi-square test was used to compare the prevalence of pterygium among different groups. The risk factors for pterygium were compared among different subgroups using univariate logistic regression analysis. Multivariate logistic regression models were used to determine independent associated risk factors. Odds ratios (OR) and 95% CI were presented. All statistical analyses were conducted using SPSS (Statistical Package for Social Sciences Inc., Chicago, IL). *P* value less than 0.05 was considered statistically significant.

## 3. Results

Out of 19,583 enumerated eligible residents, 1,767 residents were excluded because of absence at the time of examination, and 17,816 (7,803 (43.80%) men and 10,013 (56.20%) women) residents took part in the study and their results were valid. The overall response rate was 90.98%. Age of participants ranged from 50 to 101 years (62.4 ± 9.4). The distribution of age and gender in different counties is shown in Table 1.

Pterygium was diagnosed in 1,876 people, either unilateral (1,083) or bilateral (793), which is equivalent to a prevalence of 10.53% (95% CI 10.08–10.98) (Table 2). The prevalence of unilateral pterygium was higher than that of the bilateral (*P* < 0.01). In unilateral pterygium, there was no significant difference of prevalence between right and left eye (*P* = 0.40). Eight hundred and nine men (prevalence 10.37%, 95% CI 9.69–11.05) and 1,067 women (prevalence 10.66%, 95% CI 10.06–11.26) were diagnosed with pterygium. There was no significant difference of prevalence between gender groups (*P* = 0.53). The prevalence of pterygium increased statistically significantly with older age (*P* < 0.01). Among four counties selected for this study, the prevalence of pterygium in Rushan County and Huaiyin District was higher than the other two counties (*P* < 0.05). Of 1,876 people with pterygium, only 21 people had received pterygium excision surgery. The surgery rate was 1.12%.

The pterygium prevalence was compared across different subgroups using univariate analysis. The output showed that older people (*P* < 0.001) and those with outdoor time equal to or more than four hours per day (*P* < 0.001) tended to have a higher pterygium prevalence; people with a higher educational level (*P* = 0.001) and those who wore a hat and/or sunglasses when outdoors (*P* < 0.001) were more likely to have a lower prevalence. The output also showed that there was a statistical difference between the pterygium prevalence in different study counties (*P* = 0.012). No association was found with alcohol intake, smoking, or chronic systemic diseases such as diabetes, hypertension, hyperlipidemia, and cardiac diseases (Table 3).

On the basis of univariate analysis, we performed a multivariate logistic regression analysis. The presence of pterygium was set as a dependent variable and all of the characteristics that showed significant association with pterygium in the univariate analysis were set as the independent variables. Pterygium was independently associated with older age (*P* < 0.001) and outdoor time equal to or more than four hours per day (OR 1.328, 95% CI 1.182–1.491); higher educational level (OR 0.833, 95% CI 0.733–0.945) and use of hat and/or sunglasses (OR 0.107, 95% CI 0.053–0.216) were protective factors for pterygium (Table 4).

## 4. Discussion

In 1987, a population-based, cross-sectional study of visual impairment and blindness in older adults in rural Shandong Province was performed and the prevalence of pterygium was not reported [29]. To our knowledge, this survey was the first prospective population-based prevalence study of pterygium in Shandong Province. Pterygium was diagnosed in 10.53% of the rural aged population in Shandong. This prevalence was higher than that in a Beijing study which investigated 37,067 suburban adults aged 55–85 years with a prevalence of 3.76% [5]. The prevalence in the present study was also higher than that in a Handan Eye Study which described the prevalence of pterygium in a rural Chinese population aged 40 years or above was 7.1% [9]. The Handan Study chose a similar rural population as the present study; however, the difference in age and sex distribution as well as geographic locations between Handan and Shandong may account for the different prevalence. The pterygium prevalence in Shandong was lower than that in Henan County of Mongolian (17.9%) [7] and Zeku County of Tibetans (14.49%) [8] which are at the high altitude in China and in Doumen County (33.01%) [10] which is located in the south of China with a typical subtropical climate. The prevalence of pterygium varied with races and geographic locations worldwide (Table 5) [12–26]. Cameron and others [30, 31] put forward an idea of “pterygium belt”** (**located at 37 degrees north and south latitude of the equator) within which the prevalence of pterygium increased due to greater UVR exposure, but strong evidence proved the hypothesis to be oversimplistic [20]. It should be noted that the prevalence of pterygium was not only associated with geographic latitude but also related to differences in regions, study sample size, ethnic composition, age distribution, lifestyle, and occupational status [17]. In addition, the genetic difference of sensitivity to UVR should also be taken into consideration.

The results of this survey were in accordance with the idea that the prevalence of pterygium increased with age and it may be attributed to the accumulated UVR exposure. In this survey, participants aged between 70 and 79 years had the highest prevalence of 13.11%, followed by the group of 80 years or above with a prevalence of 11.72%. The slightly lower pterygium prevalence in participants aged 80 years or above may be ascribed to a smaller sample size of 1,041 people. Fotouhi investigated 4,564 people in Tehran and found that the prevalence of pterygium increased from 0.1% in people aged 1–19 years to 7.8% in those aged 60 years or above [13]. The study in Indonesia [26] described a statistically significant increase of the prevalence in different age groups, from 2.9% in people aged 21–29 years to 17.3% in those older than 50 years.

The idea that gender is an associated factor for pterygium is controversial. Studies in Doumen County [10] showed that women have a higher risk than men. In contrast, others found that men are at significantly higher risk than women [15, 17, 20, 23, 25, 32]. In the present study, there was no significant difference in the prevalence of pterygium between men and women, which was similar to the results of the study performed in south India [21]. We believe that the lifestyle in the rural area in Shandong may account for this result. In rural areas within the province, the majority of residents aged 50 or above take part in outdoor farming work and agricultural income is the exclusive economic source for rural families. In order to improve their economic status, women have to do outdoor farming work rather than staying at home.

In the present study, outdoor time equal to or more than four hours per day during daylight had a positive association with pterygium; the longer the subjects stayed outside, the higher the prevalence was. A large number of participants were occupied with agricultural or fishing activities and they spent most of their day time outdoors. Consequently, cumulative exposure to UVR increased the prevalence of pterygium. Simultaneously, we proved that using hat and/or sunglasses was protective for pterygium, which agreed with the conclusions of the Barbados Eye Study [14] and Rosenthal [33]. The mechanism behind this phenomenon could be due to the blocking of ocular UVR exposure and other environmental factors such as dust [31]. Therefore, people using hat or sunglasses had lower prevalence of pterygium.

Previous surveys for pterygium had reported that there was relation between educational level and pterygium [14, 19, 21, 23]. Researchers considered that higher level of education played a protective role in the development of pterygium. Tan et al. [34] investigated the Chesapeake Bay watermen in the United States and found that more than eight years of education was beneficial in preventing pterygium. Similarly, in our study, data indicated that the pterygium prevalence was higher in people whose educational level was less than six years. Higher education may lead to qualified jobs and superior socioeconomic status; therefore, those subjects may not spend most of their time working outdoors. Furthermore, people with higher education may have strong self-protection awareness to use a hat or sunglasses when they are engaged in outdoor work.

In this population-based study, alcohol intake, smoking, and chronic systemic diseases such as diabetes, hypertension, hyperlipidemia, and cardiac diseases were introduced in the logistic regression model. However, we found that there was no association between these factors and pterygium, a result which differed from other surveys. Some studies showed that there was a negative correlation between smoking and pterygium by a possible mechanism of promoting carcinogenesis, upregulating cytokines, and proteins which were responsible for cell proliferation and migration [14, 15, 25]. Conversely, Wong found that smoking was a risk factor for pterygium in the Chinese population in Singapore [20]. It is interesting to note that West found there was a slight protective effect between pterygium and diabetes [25]. Additionally, the Singapore Malay Eye Study [12] reported that there were weak associations between pterygium and systolic blood pressure and total cholesterol levels. Therefore, further study is needed to determine whether these factors have effects on pterygium occurrence.

The strengths of our study included the characteristics of a population-based approach, random sampling, large sample size, and high response rate, which guaranteed the representativeness of this survey. However, there were some limitations in our study. First, the location and classification of the severity of pterygium were not recorded during the survey. Second, UVR exposure, which may be the main cause for pterygium, was estimated by outdoor time in a questionnaire, rather than an objective measurement. Finally, not all the refractive statuses of participants were available in this study; only those presenting with VA ≤ 0.5 in either eye were examined by an autorefraction test. Therefore, the relationship between pterygium and astigmatism was unsure.

In conclusion, the prevalence of pterygium was 10.53% (95% CI 10.08, 10.98) among rural people aged 50 years or above in Shandong Province, China. Pterygium was positively associated with older age and outdoor time equal to or more than four hours per day and negatively associated with educational level and use of hat and/or sunglasses. Because severe pterygium can result in visual impairment and blindness, it is important to take some preventive measures to diminish the prevalence of pterygium, such as suggesting people wear a hat and/or sunglasses whenever they are outside in the sunshine, educating farmers to raise their awareness for pterygium, and providing surgery service when pterygium is diagnosed. We hope that every effort will be taken to avoid the blindness caused by severe pterygium.

## Figures and Tables

**Table 1:** Distribution of participants by age, gender, and examination site (number (%)).

Participants		Age groups (years)				Gender groups	
Area	Number	50–59	60–69	70–79	≥80	Male	Female
Rushan	4994	2138 (42.81)	1376 (27.55)	1077 (21.57)	403 (8.07)	2169 (43.43)	2825 (56.57)
Huaiyin	3040	1721 (56.61)	727 (23.91)	427 (14.05)	165 (5.43)	1374 (45.20)	1666 (54.80)
Tengzhou	4916	2293 (46.64)	1357 (27.60)	1006 (20.46)	260 (5.29)	2183 (44.40)	2733 (55.60)
Juancheng	4866	2271 (46.67)	1727 (35.49)	655 (13.46)	213 (4.38)	2077 (42.68)	2789 (57.32)
Total	**17816**	**8423 (47.28)**	**5187 (29.11)**	**3165 (17.76)**	**1041 (5.84)**	**7803 (43.80)**	**10013 (56.20)**

**Table 2:** Prevalence of pterygium in participants by age, gender, and examination site.

	Participants number (%)	Pterygium number	Pterygium prevalence (%) (95% CI)	*P* value
Age (years)				
50–59	8423 (47.28)	732	8.69 (8.09–9.29)	0.00^a^
60–69	5187 (29.12)	607	11.70 (10.83–12.57)	
70–79	3165 (17.76)	415	13.11 (11.93–14.29)	
≥80	1041 (5.84)	122	11.72 (9.77–13.67)	
Gender				
Male	7803 (43.80)	809	10.37 (9.69–11.05)	0.53^b^
Female	10013 (56.20)	1067	10.66 (10.06–11.26)	
Area				
Rushan	4994 (28.03)	558	11.17 (10.30–12.04)	0.01^c^
Huaiyin	3040 (17.07)	341	11.22 (10.10–12.34)	
Tengzhou	4916 (27.59)	522	10.62 (9.76–11.48)	
Juancheng	4866 (27.31)	455	9.35 (8.53–10.17)	
Total	**17816 (100.00)**	**1876**	**10.53 (10.08**–**10.98)**	

^a^ *P* value between different age groups by *χ* ^2^ test.

^b^ *P* value between male and female by *χ* ^2^ test.

^c^ *P* value of comparison between four examination sites by *χ* ^2^ test.

**Table 3:** Univariate analysis of the risk factors for pterygium.

Risk factors	Participants number	Pterygium number	Odds ratio (95% CI)	*P* value
Age (years)				
50–59	8423	732	1.0	**0.000**
60–69	5187	607	1.392 (1.243–1.560)	
70–79	3165	415	1.586 (1.395–1.802)	
≥80	1041	122	1.395 (1.138–1.710)	
Sex				
Male	7803	809	1.0	0.534
Female	10013	1067	1.031 (0.936–1.136)	
Areas				
Rushan	4994	558	1.0	**0.012**
Huaiyin	3040	341	1.004 (0.871–1.159)	
Tengzhou	4916	522	0.944 (0.832–1.072)	
Juancheng	4866	455	0.820 (0.720–0.934)	
Education				
Primary school and below	13519	1481	1.0	**0.001**
Junior school and above	4297	395	0.823 (0.732–0.925)	
Outdoor time (hours per day)				
<4	6682	795	1.0	**0.000**
≥4	11134	1081	1.256 (1.140–1.384)	
Wearing hat and/or sunglasses				
No	17,177	1,868	1.0	**0.000**
Yes	639	8	0.104 (0.052–0.209)	
Alcohol intake				
Never	12,785	1,345	1.0	0.989
Current	4,330	456	1.001 (0.895–1.120)	
Past	701	75	1.019 (0.797–1.303)	
Smoker				
Never	12,908	1,354	1.0	0.811
Current	4,257	457	1.026 (0.917–1.148)	
Past	651	65	0.947 (0.728–1.230)	
Diabetes				
No	17,213	1,823	1.0	0.157
Yes	603	53	0.814 (0.611–1.083)	
Hypertension				
No	15,196	1,592	1.0	0.576
Yes	2,620	284	1.039 (0.909–1.187)	
Hyperlipidemia				
No	17,229	1,820	1.0	0.427
Yes	587	56	0.893 (0.675–1.181)	
Cardiac diseases				
No	16,766	1,759	1.0	0.505
Yes	1,050	117	1.070 (0.877–1.305)	

**Table 4:** Multivariate model of the risk factors for pterygium.

Risk factors	OR_adj_ (95% CI)	*P*-value
Age (years)		
50–59	1.0	0.000
60–69	1.425 (1.270–1.599)	
70–79	1.610 (1.409–1.840)	
≥80	1.466 (1.189–1.807)	
Areas		
Rushan	1.0	0.032
Huaiyin	1.078 (0.931–1.247)	
Tengzhou	0.867 (0.759–0.989)	
Juancheng	0.989 (0.857–1.141)	
Education (years)		
Primary school and below	1.0	0.005
Junior school and above	0.833 (0.733–0.945)	
Outdoor time (hours per day)		
<4	1.0	0.000
≥4	1.328 (1.182–1.491)	
Wear sunglasses or hats		
No	1.0	0.000
Yes	0.107 (0.053–0.216)	

**Table 5:** The prevalence of pterygium in different regions of the world.

Country	Year	Age (yr)	Prevalence of pterygium
Beijing (aged rural population) [5]	2010	55–85	3.76%
Beijing (rural and urban areas of Beijing) [6]	2007	≥40	2.88%
Henan County, China (Mongolian population) [7]	2009	≥40	17.9%
Tibetans, China [8]	2007	≥40	14.49%
Handan, China (rural adult population) [9]	2013	≥30	6.0%
Doumen County, China [10]	2002	≥50	33.01%
Singapore (adult Malay population) [12]	2010	40–79	12.3%
Tehran, Iran [13]	2008	≥60	7.8%
Barbados (black subjects) [14]	2001	40~84	23.7%
Victoria, Australia [15]	2000	≥40	2.83%
Korea [16]	2008–2010	≥30	6.7%
South-western Japan [17]	2009	≥40	30.8%
Myanmar [18]	2008	≥40	19.6%
O Salnes, Spain [19]	2010	≥40	5.9%
Singapore [20]	2012	40–79	6.9%
South India [21]	2013	≥30	11.7%
Central India (rural population) [22]	2013	≥30	12.91%
Singapore (Malays, Indians, and Chinese) [23]	2012	≥40	10.1%
Northern Japan [24]	2013	40–74	4.4%
Latinos [25]	2009	≥40	16.2%
Indonesia [26]	2002	≥21	10.0%
